# A Highly Versatile X-ray and Electron Beam Diamond Dosimeter for Radiation Therapy and Protection

**DOI:** 10.3390/ma16020824

**Published:** 2023-01-14

**Authors:** Sara Pettinato, Marco Girolami, Antonella Stravato, Valerio Serpente, Daniela Musio, Maria C. Rossi, Daniele M. Trucchi, Riccardo Olivieri, Stefano Salvatori

**Affiliations:** 1 Engineering Faculty, Niccolò Cusano University, Via Don Carlo Gnocchi 3, 00166 Rome, Italy; 2 Istituto di Struttura della Materia, Consiglio Nazionale delle Ricerche (ISM-CNR), DiaTHEMA Lab, Strada Provinciale 35D, 9, Montelibretti, 00010 Rome, Italy; 3 Azienda Ospedaliera “San Giovanni–Addolorata”, Via dell’Amba Aradam, 8, 00184 Rome, Italy; 4 Department of Industrial, Electronic and Mechanical Engineering, Università degli Studi Roma Tre, Via Vito Volterra 62, 00146 Rome, Italy

**Keywords:** radiation therapy, radiation protection, dosimetry, CVD diamond detectors, electron beams, X-rays

## Abstract

Radiotherapy is now recognized as a pillar in the fight against cancer. Two different types are currently used in clinical practice: (1) external beam radiotherapy, using high-energy X-rays or electron beams, both in the MeV-range, and (2) intraoperative radiotherapy, using low-energy X-rays (up to 50 keV) and MeV-range electron beams. Versatile detectors able to measure the radiation dose independently from the radiation nature and energy are therefore extremely appealing to medical physicists. In this work, a dosimeter based on a high-quality single-crystal synthetic diamond sample was designed, fabricated and characterized under low-energy X-rays, as well as under high-energy pulsed X-rays and electron beams, demonstrating excellent linearity with radiation dose and dose-rate. Detector sensitivity was measured to be 0.299 ± 0.002 µC/Gy under 6 MeV X-ray photons, and 0.298 ± 0.004 µC/Gy under 6 MeV electrons, highlighting that the response of the diamond dosimeter is independent of the radiation nature. Moreover, in the case of low-energy X-rays, an extremely low limit of detection (23 nGy/s) was evaluated, pointing out the suitability of the device to radiation protection dosimetry.

## 1. Introduction

Radiation therapy (RT) is a well-established physical therapy for cancer treatment, using high-energy ionizing radiations, such as X-rays or particle beams, to destroy or shrink cancer cells and tumors [1].

Different RT techniques have been introduced and proposed over the years. Currently, the most commonly used are External Beam RT (EBRT) and Intra-Operative RT (IORT). The first difference between the two techniques is that in EBRT the patient is treated in several sessions, whereas in IORT the radiation dose is delivered all at once during surgery [2]. In addition, in EBRT the therapeutic effect is desired for deep-tissue tumors, therefore radiation with energies in the order of MeV (i.e., with sufficient energy to avoid significant attenuation by surface tissue) are required. For this reason, EBRT operates with linear accelerators (LINACs) producing pulsed radiation beams (electrons or X-rays) with energies in the 4–25 MeV range. Conversely, IORT is used for surface-tissue tumors (i.e., requiring radiations with a low penetration depth), and that is the reason why special mobile LINACs are employed, able to deliver low-energy X-rays (up to 50 keV) [3,4], as well as electron beams in the 4–6 MeV range.

In both cases, as in all RT techniques, the goal is two-fold: (1) cancer treatment; (2) minimization of the radiation dose delivered to the healthy tissue surrounding the tumor. This is particularly challenging in EBRT, which always requires high penetration depths. In recent years, in order to preserve healthy tissue even with EBRT treatments, two techniques have become widespread: Intensity Modulated RT (IMRT) [5] and Volumetric Arc RT (VMAT) [6], providing conformal dose distributions by the use of small fields, high dose gradients and space-time modulation of the radiation beam.

As can be easily deduced, regardless of the type of RT, the quality of treatment is of the greatest importance, and accurate dose delivery is a key point. In particular, the precise monitoring of the radiation dose delivered to the patient during cancer treatments, as well as of the dose-rate, is crucial. In this context, the use of accurate dosimeters is mandatory for the calibration of the measurement system, as well as for the validation of the treatment plan. The International Atomic Energy Agency (IAEA) [7] defines the following requirements for a dosimeter to be used for RT: stability under irradiation, settling time in the dark-to-light transient (i.e., high response speed), repeatability, sensitivity (i.e., measurable collected charge per unit of administered dose), and linearity with both dose and dose-rate. Diamond detectors for ionizing radiation effectively meet these requirements. Indeed, in the past two decades, diamond has been demonstrated to be the material of choice for the fabrication of high-performance dosimeters for RT, thanks to its special features: response speed in the nanosecond range [8,9,10], radiation hardness [11], high sensitivity and linearity with dose-rate [12], and, most of all, tissue-equivalence [13], implying that no correction factors are needed when quantifying the dose absorbed by human tissue. High-quality “electronic-grade” diamond samples with an impurity concentration three orders of magnitude lower than natural diamond are now available at a relatively low cost, thanks to the optimization of the chemical vapor deposition (CVD) technique. Dosimeters based on CVD diamond are, indeed, widely used for EBRT [14,15,16,17,18] and IORT [19,20,21], as well as for soft X-rays [22,23,24], UV light [25,26], charged particles [27,28,29], and neutrons [30,31,32].

However, the features of the diamond detector (e.g., thickness, type and geometry of the metal contacts, operation in photovoltaic or photoconductive mode) are usually tailored to the specific radiation being monitored, aimed at optimizing the detector performance. Of course, a unique multi-purpose dosimeter able to offer a good performance with radiations of different nature and energy range would be highly desirable, especially in the RT field. For this purpose, we demonstrate in this work that a dosimeter based on a state-of-the-art single-crystal chemical-vapor-deposition (CVD) diamond sample ensures excellent sensitivity and linearity with all the radiations of interest for EBRT and IORT. More specifically, the dosimeter was extensively characterized under: (1) low-energy (keV range) X-rays generated by an X-ray tube; (2) high-energy (MeV range) X-rays; and (3) high-energy (MeV range) electrons generated by a medical LINAC. In addition, in the case of low-energy X-rays, a limit of detection of only 23 nGy/s was evaluated, pointing out the suitability of the device for radiation protection dosimetry.

## 2. Materials and Methods

**Figure 1:**
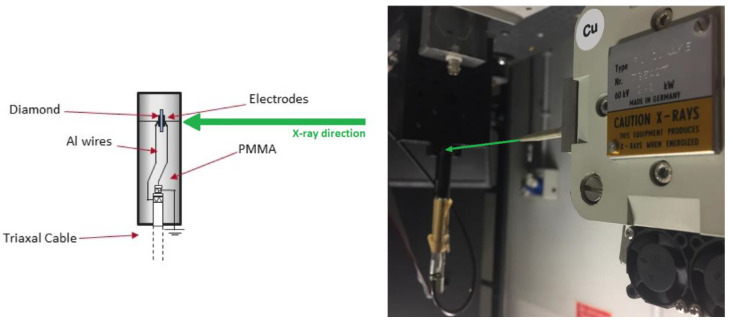
Cross-sectional scheme of the fabricated dosimeter (**left**); Setup of the characterization under continuous low-energy X-rays (**right**), showing on the right the Coolidge tube (Cu target) with the output collimator pointed towards the dosimeter. Green arrows indicate the direction of the X-ray beam focused on the active area of the dosimeter.

A 4.5 × 4.5 × 0.5 mm^3^ “electronic-grade” single-crystal CVD diamond sample (Element Six Ltd., Oxfordshire, UK) was used as the active medium for the fabrication of the dosimeter. Aimed at removing possible non-diamond phases, organic contaminants and particulate from all the surfaces of the sample, the following cleaning procedure was adopted: (1) acid cleaning in a H_2_SO_4_:HClO_4_:HNO_3_ (1:1:1) mixture for 30 min at boiling point; (2) ultrasonic bath in hot acetone for 5 min; (3) rinsing in deionized water; and (4) drying in pure nitrogen flow. Then, two 300 nm-thick Ag contacts were fabricated on the top and bottom surfaces of the sample by means of two subsequent sputtering depositions, resulting in a typical MSM (metal-semiconductor-metal) structure working in photoconductive mode. The contact geometry (a circular pad with a radius of 1.6 mm) was automatically defined with a stainless-steel shadow mask, with no need for photolithographic steps. Therefore, the active volume of the MSM structure was around 4 mm^3^. To ensure mechanical stability, the sample was fixed with epoxy resin (Epotek^®^ 301, REMAK S.r.l., Milan, Italy) into a circular Rexolite^®^ ring. Two thin Al wires were glued on the sample contacts with silver paste (G3790, Agar Scientific Ltd., Stansted, Essex, UK) and then soldered to the outer shield and the inner conductor, respectively, of a 3.5 m long triaxial cable for detector biasing and signal collection. The structure was then encapsulated in a polymethyl-methacrylate (PMMA) cylindrical housing, 7.4 mm in diameter and 40 mm-long, with the triaxial cable inserted into a stainless-steel cylinder. The PMMA cylinder was finally filled with Epotek^®^ 301 epoxy resin and coated with black acrylic paint, thus ensuring waterproofing to the dosimeter and eliminating any effect of the ambient light. A cross-sectional scheme of the fabricated dosimeter is shown in Figure 1 (left).

## 3. Results and Discussion

### 3.1. Characterization under Low-Energy Continuous X-rays

The performance of the assembled dosimeter was investigated under continuous low-energy X-rays by means of a Coolidge tube equipped with a Cu target (Seifert Electronic GmbH, Ennepetal, Germany). No filter was interposed between the X-ray source and the dosimeter. In this way, in addition to the 8.05 keV (K_α_) and 8.91 keV (K_β_) lines emitted by the Cu target, the Bremsstrahlung radiation was absorbed by the detector. The detector was placed at a distance of 12 cm from the output slit of a collimator (1 mm in diameter), resulting in a circular spot-size on the active area of about 2.5 mm^2^, and then connected to a Keithley 487 electrometer (Keithley Instruments, Solon, OH, USA) simultaneously used as a voltage supply (bias voltage was set to 10 V) and a current meter. The setup is shown in Figure 1 (right).

**Figure 2:**
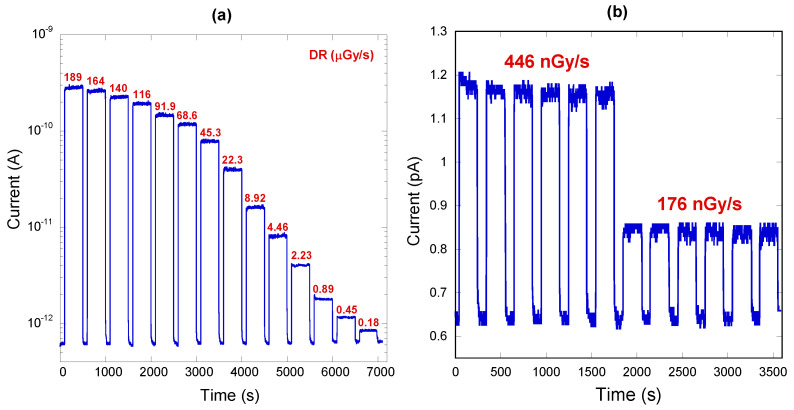
(**a**) Current measured under low-energy X-rays as a function of time. “X-ray on” and “X-ray off” periods were set to 300 s and 100 s, respectively. Red numbers refer to dose-rate values. Current values are reported on a log scale to better highlight the signals at very low dose-rates (<1 µGy/s); (**b**) Current measured under the two lowest investigated dose-rates: six subsequent “X-ray on” and “X-ray off” periods (200 s and 100 s, respectively) were acquired for each dose-rate.

For the tests, the tube acceleration voltage was set to 40 kV, while the current was varied in the range 0.05–40 mA, resulting in an X-ray dose-rate (*DR*) varying in the range 0.18–189 µGy/s, as measured with a reference ionization chamber (Farmer mod. NE 2536/3C, Thermo Fisher Scientific Inc., Waltham, MA, USA) connected to a commercial dosimeter (Farmer mod. NE 2670, Thermo Fisher Scientific, Waltham, MA, USA). Results are reported in Figure 2. Firstly, it appears clear from Figure 2a that experimental data highlight a very fast response of the device, with both the rise and fall times lower than 0.5 s, representing the delay time between two consecutive measurement points. Most significantly, at a given dose-rate, the current signal reaches its stationary value almost immediately, showing no priming effects [33,34].

In the final part of the measurement session, aimed at verifying the reproducibility and stability of the signal, the detector was repeatedly tested under the two lowest investigated dose-rates (446 nGy/s and 176 nGy/s) for about 1 h. As can be seen from Figure 2b, the current signal was perfectly reproducible and stable at each “X-ray on” period, with no appreciable drifts from its average value.

**Figure 3:**
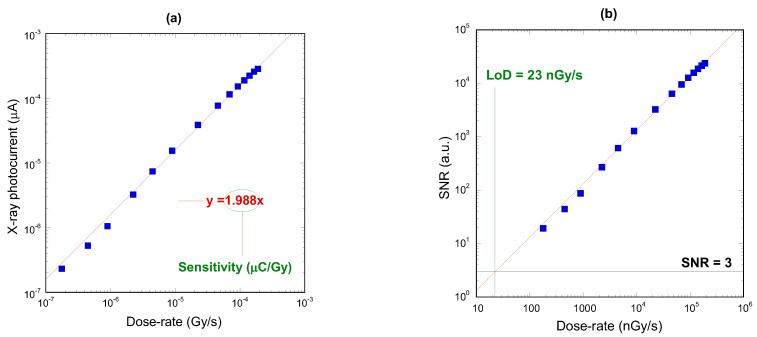
(**a**) Average X-ray photocurrent values as a function of the dose-rate for low-energy X-ray characterizations. Red line indicates the linear fitting curve (Δ = 1). Error bars, calculated as the standard deviation of the X-ray photocurrent, are smaller than the symbols; (**b**) Signal-to-noise ratio as a function of the dose-rate. The limit of detection is evaluated from the intercept of the linear fitting curve (red line) with the horizontal line corresponding to *SNR* = 3.

Figure 3a reports the average X-ray photocurrent values as a function of the dose-rate. X-ray photocurrent *I_ph_* was calculated as *I_ph_* = *I_tot_ − I_d_,* where *I_tot_* is the total current acquired during the “X-ray on” periods and *I_d_* is the dark current measured during the “X-ray off” periods. Data perfectly follow Fowler’s law [35] for electric conductivity of solid-state detectors, according to which *I_ph_* ∝ *DR*^Δ^, where Δ is the linearity coefficient. Values of Δ around unity are always desirable, even mandatory in case of dosimeters for radiotherapy. Here, the best fit returned Δ ≈ 0.957 with a correlation coefficient equal to *R* = 0.99942, denoting a very good linearity with *DR*. This allowed us to estimate with a reasonable accuracy the sensitivity of the detector, evaluated from the slope of the linear fitting curve (Δ = 1) to be 1.988 ± 0.005 µC/Gy.

It was then of interest to evaluate the limit of detection (*LoD*) of the fabricated dosimeter, which is an important figure of merit for devices operating at very low dose-rates, as dosimeters for radiation protection. As defined by the IUPAC (International Union of Pure and Applied Chemistry) the *LoD* is the dose-rate corresponding to a signal-to-noise ratio *SNR* = 3, i.e., it is the lowest dose-rate at which there is a measurable signal 3 times larger than the noise level [36]. *SNR* can be calculated as the ratio between the X-ray average photocurrent measured at each dose-rate value and the standard deviation of the dark current, as reported in the following equation:

(1) $$SNR=\frac{\frac{1}{n}\sum_{i}^{n}I_{ph_{i}}}{\sqrt{\frac{1}{n}\sum_{i}^{n}{\left(I_{d}{}_{i}-{\bar{I}}_{d}\right)}^{2}}}$$

where *n* is the number of acquisitions of the “X-ray on” (“X-ray off”) period for the numerator (denominator), and ${\bar{I}}_{d}$ is the average dark current. As can be seen from Figure 3b, showing *SNR* as a function of the dose-rate, the evaluated *LoD* is only 23 nGy/s, which is remarkably comparable to the *LoD* values recently reported for state-of-the-art X-ray direct detectors based on organic materials [37] and perovskites [38]. To our knowledge, this is also the first time that *LoD* has been evaluated for a diamond-based X-ray dosimeter, and its suitability highlighted for radiation protection applications.

### 3.2. Characterization under High-Energy Pulsed X-rays

**Figure 4:**
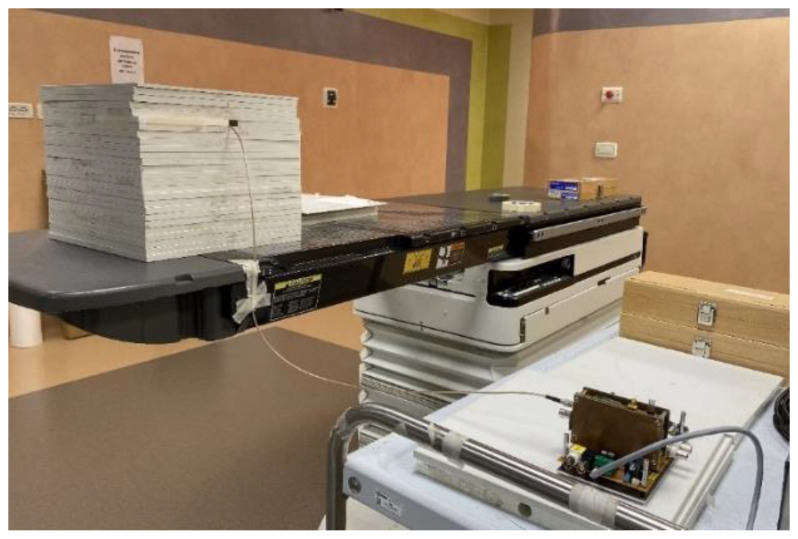
Picture of the characterization setup under high-energy pulsed X-rays. The dosimeter, placed inside a solid Plexiglass^®^ phantom (on the left), is positioned at the isocenter of LINAC, under a 10 × 10 cm^2^ field.

After the characterization under low-energy continuous X-rays, the dosimeter was tested under high-energy pulsed X-rays produced by a medical LINAC (Clinac iX, Varian Inc) installed at the Radiation Therapy Department of “San Giovanni Addolorata” hospital in Rome (Italy). The LINAC acceleration voltage was set to 6 MV, in order to produce X-ray photons with energy up to 6 MeV. Measurements were performed under standard conditions, i.e., the dosimeter was inserted into a water-equivalent Plexiglass^®^ phantom, to assure electronic equilibrium [39], as shown in Figure 4. The dosimeter was placed at the LINAC isocenter, at a source-to-surface distance of 100 cm, and under a 10 × 10 cm^2^ field. The dosimeter, immersed at a depth of 5 cm, was oriented perpendicular to the direction of the impinging X-rays, and biased at 10 V, as in the case of the characterization under low-energy X-rays. A specifically developed front-end/readout electronics, described in detail elsewhere [40,41], was used, allowing for pulse-by-pulse measurement of the photocurrent signal generated by the incident photons. Before taking the measurements, a proper calibration procedure of the front-end electronics was carried out by emulating the photocurrent pulsed response of the detector with a Keithley 6221 programmable current source (Keithley Instruments, Solon, OH, USA), thus evaluating the transfer function (i.e., the analog-to-digital output code as a function of the input charge) of the instrument. A detailed description of the calibration procedure can be found in [41].

**Figure 5:**
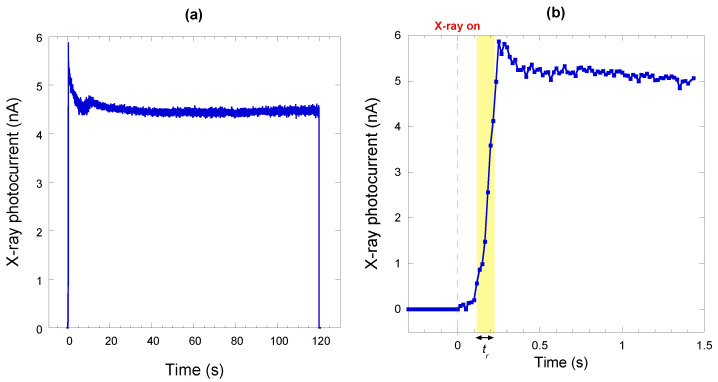
(**a**) X-ray photocurrent signal acquired at a fixed *DR* of 1 Gy/min. The total acquisition time (120 s) corresponds to the acquisition of 7200 X-ray pulses; (**b**) Zoomed image of the X-ray photocurrent signal soon after the X-rays are switched on. Yellow box extends from 10% to 90% of the final photocurrent value to highlight the rise time *t_r_*.

Figure 5a shows an example of an X-ray photocurrent signal acquired by setting the LINAC to deliver a total dose of 2 Gy at *DR* = 1 Gy/min. At this *DR* value, the pulse repetition frequency is equal to 60 Hz, therefore the acquisition period of 120 s allowed for the collection of 7200 X-ray pulses. As can be seen, the implemented detection system was able to monitor the pulse intensity in real time. Indeed, the system clearly displays the overshoot of the photocurrent signal (i.e., of the delivered dose) at the beginning of the emission, allowing for the perfect tracking of the procedure executed by the LINAC to deliver the desired total dose.

In addition, the dedicated front-end electronics used for pulsed measurements allowed for a more accurate definition of the signal rise-time (defined as the time required for the signal to rise from 10% to 90% of its stationary value), which was evaluated to be *t_r_* ≈ 100 ms, as can be inferred from Figure 5b. However, it is worth stressing here that the signal rise time is not limited by the response speed of the diamond dosimeter, but rather by the LINAC transient at the start of the beam. The actual rise time of the diamond detector alone is, indeed, in the nanosecond range, as already reported in the literature on devices fabricated with similar “electronic-grade” samples tested under ultra-fast sources such as excimer lasers [10] or radioactive sources [32].

**Figure 6:**
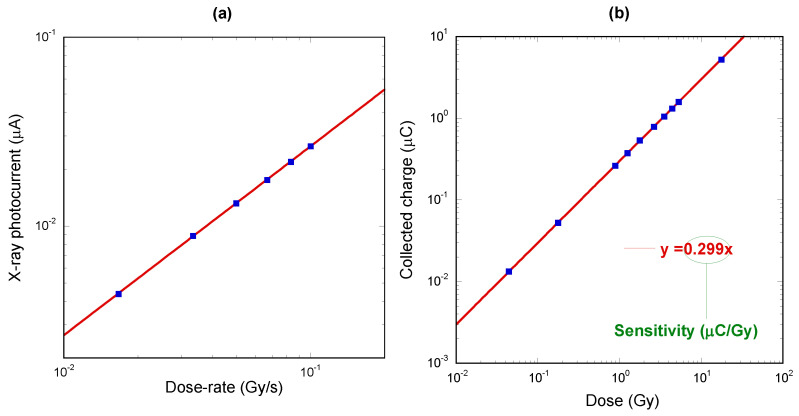
(**a**) Average X-ray photocurrent values as a function of the dose-rate for high-energy X-ray characterizations. Red line indicates the linear fitting curve (Δ = 1). Error bars, calculated as the standard deviation of the X-ray photocurrent, are smaller than the symbols; (**b**) Total collected charge as a function of the radiation dose acquired for high-energy X-ray characterizations. Red line indicates the linear fitting curve (Δ = 1). The delivered dose was measured by the reference ionization chamber.

The same measurements were repeated at all available dose-rates of the LINAC system, in the range 1–6 Gy/min. For each dose-rate, the LINAC was always set to release a total dose of 2 Gy. Results are reported in Figure 6a, showing the average X-ray photocurrent values as a function of the dose-rate. As in the case of low-energy X-rays (Figure 3a), data again follow Fowler’s law, but here the linearity with dose-rate is almost perfect (Δ = 1.0003) with *R* = 0.99994. The slope of the linear fitting curve was evaluated to be 0.264 ± 0.002 µC/Gy. However, it is worth observing that this value cannot be interpreted as the sensitivity of the dosimeter, because it does not consider the X-ray attenuation due to the 5 cm-thick Plexiglass^®^ phantom. The effective sensitivity of the dosimeter under 6 MeV X-ray photons was successively inferred by means of dose measurements performed with an ionization chamber (FC65-G, IBA Dosimetry, calibrated at the National Institute of Ionizing Radiation Metrology, ENEA-INMRI, Rome, Italy) connected to the precision electrometer Dose-1 (IBA Dosimetry GmbH, Schwarzenbruck, Germany), and inserted into the solid phantom at the same position as the diamond detector. The LINAC was set to produce 6 MeV X-rays in the dose range 0.1–10 Gy, representing the dose values used routinely during clinical treatments. The dose-rate was kept constant at 3 Gy/min. The dose absorbed by the solid phantom was calculated by applying the calibration factors and corrections for the influence quantities (i.e., pressure, temperature, polarity effect, and ion recombination), according to the IAEA TRS398 protocol [7], as well as by considering the dose-to-water cross-calibration coefficients. In this way, a calibration chart was obtained relating the specific dose at a depth of 5 cm to the nominal dose sourced by the LINAC. The same experiment was then repeated with the diamond detector itself, but in this case giving full consideration to the attenuation caused by the Plexiglass^®^ phantom. The dose delivered to the sample was varied according to the dose calibration procedure performed with the ionization chamber. Moreover, the dedicated front-end electronics was set to operate in charge-mode, i.e., allowing for the measurement of the total charge collected during a single acquisition period.

The experimental results are reported in Figure 6b, again showing an excellent linearity with dose in the investigated range. The slope of the linear fitting curve, factoring in the radiation attenuation operated by the phantom, can now be used to obtain the real detector sensitivity under 6 MeV X-ray photons, which is 0.299 ± 0.002 µC/Gy. It is worth highlighting that this value is about 0.15 times the sensitivity value estimated with low-energy X-rays (1.988 ± 0.005 µC/Gy), in agreement with the ratio between the corresponding mass attenuation coefficients of X-rays in carbon (0.03 cm^2^/g at 6 MeV, and 0.2 cm^2^/g at 40 keV) [42].

### 3.3. Characterization under High-Energy Pulsed Electron Beams

**Figure 7:**
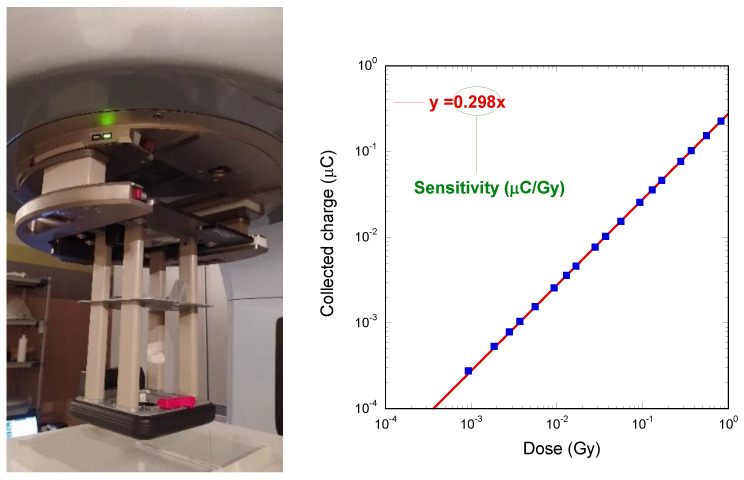
(**left**) The electron applicator used for characterizations under electron beams. The detector, inserted into a 3 cm thick Plexiglas^®^ slab at the LINAC isocenter, is visible on the bottom; (**right**) Total collected charge as a function of the dose acquired for high-energy pulsed electron beams.

A final characterization was carried out to evaluate the dosimeter response to high-energy electron beams typically employed in radiation therapy treatments of superficial tumors [43]. Pulsed electron beams can be directly obtained by the Clinac iX system by simply removing the W target (producing X-rays) from the LINAC head. The dosimeter was again placed at the LINAC isocenter, and inserted into a 3 cm thick Plexiglas^®^ phantom to assure electronic equilibrium. It is worth noting here that the phantom thickness is lower than that used for the X-ray experiments, due to the lower penetration depth of the electrons with respect to X-rays with the same energy. A 15 × 15 cm^2^ electron applicator, as shown in Figure 7, was attached to the LINAC head to ensure beam convergence on the dosimeter.

Measurements were performed under 6 MeV electrons irradiation at a fixed dose-rate of 10 Gy/min. The dose was varied in the range 0.001–1 Gy. The dosimeter was biased at 10 V. The results are shown in Figure 7, reporting the total charge collected at each delivered dose. As in the case of 6 MeV X-rays, the linearity with dose is excellent. Sensitivity, evaluated from slope of the linear fitting curve (red line), is 0.298 ± 0.004 µC/Gy, in agreement with the value found under 6 MeV X-rays (0.299 ± 0.002 µC/Gy). By considering that for 6 MeV electrons, at a depth of 1.5 cm in a water equivalent medium, the percentage of depth dose (PDD) is equal to about 100% [44], it is possible to assume that the absorbed dose is equal to the nominal dose set for LINAC, thus justifying the same sensitivity found under 6 MeV X-rays, for the evaluation of which the actual dose values absorbed by the sample were used. The experimental results also highlight the effectiveness of diamond in measuring the absorbed dose in a tissue-equivalent medium, then qualifying the diamond dosimeter as a transfer standard device [45].

Finally, it is worth mentioning here that a preliminary characterization of the angular dependence of the diamond detector photoresponse was performed by varying the orientation of the device within a ±10° angle with respect to perpendicular direction, returning a standard deviation lower than 1%. While promising, this result must of course be confirmed with a more exact quantification of the angular dependence for a wider range of angles, to be performed in the future. Moreover, work is in progress to investigate the temperature dependence of the dosimeter photoresponse in a water phantom, under both X-ray photons and electrons with two different high-energy values (6 MeV and 18 MeV).

## 4. Conclusions

A dosimeter based on a high-quality single-crystal CVD diamond sample was fabricated and fully characterized under the radiations typically employed in modern radiotherapy techniques: low-energy (up to 40 keV) continuous X-rays; high-energy (6 MeV) pulsed X-rays; and high-energy (6 MeV) pulsed electron beams. The experimental results demonstrated excellent performance (e.g., response speed, linearity with dose and dose-rate) in all cases. In particular, it is worth highlighting three main results:

(1) The almost equal sensitivity values found under 6 MeV X-rays and 6 MeV electrons;
(2) The agreement between the ratio of the sensitivities evaluated under low- and high-energy X-rays and the ratio of the respective mass attenuation coefficients in carbon;
(3) The extremely low limit of detection found under low-energy X-rays.

All of these results denote the effectiveness of the dosimeter in monitoring the radiation dose and dose-rate independently of the beam nature and energy. Therefore, CVD diamond technology is demonstrated in this work to have reached a significantly high standard, allowing for the fabrication of extremely versatile detectors, suitable for both intra- and extra-operative radiotherapy techniques, as well as for radiation protection dosimetry.

## Data Availability

The data presented in this study are available on request from the corresponding author.
